# Prognostic factors for persistent fatigue after COVID-19: a prospective matched cohort study in primary care

**DOI:** 10.3399/BJGP.2022.0158

**Published:** 2023-02

**Authors:** Benthe H König, Cornelia HM van Jaarsveld, Erik WMA Bischoff, Henk J Schers, Peter LBJ Lucassen, Tim C Olde Hartman

**Affiliations:** Department of Primary and Community Care, Radboud Institute for Health Sciences, Radboud University Medical Center, Nijmegen, the Netherlands.; Department of Primary and Community Care, Radboud Institute for Health Sciences, Radboud University Medical Center, Nijmegen, the Netherlands.; Department of Primary and Community Care, Radboud Institute for Health Sciences, Radboud University Medical Center, Nijmegen, the Netherlands.; Department of Primary and Community Care, Radboud Institute for Health Sciences, Radboud University Medical Center, Nijmegen, the Netherlands.; Department of Primary and Community Care, Radboud Institute for Health Sciences, Radboud University Medical Center, Nijmegen, the Netherlands.; Department of Primary and Community Care, Radboud Institute for Health Sciences, Radboud University Medical Center, Nijmegen, the Netherlands.

**Keywords:** determinants, long COVID, outpatients, persistent fatigue, primary healthcare, prospective cohort study

## Abstract

**Background:**

Persistent fatigue after COVID-19 is common; however, the exact incidence and prognostic factors differ between studies. Evidence suggests that age, female sex, high body mass index, and comorbidities are risk factors for long COVID.

**Aim:**

To investigate the prevalence of persistent fatigue after COVID-19 in patients with a mild infection (managed in primary care) during the first wave of the pandemic and to determine prognostic factors for persistent fatigue.

**Design and setting:**

This was a prospective cohort study in Dutch general practice, combining online questionnaires with data from electronic health records.

**Method:**

Patients who contacted their GP between March and May 2020 and were diagnosed with COVID-19 during the first wave of the pandemic were included. Patients were matched to controls without COVID-19 based on age, sex, and GP practice. Fatigue was measured at 3, 6, and 15 months, using the Checklist of Individual Strength.

**Results:**

All the participants were GP attendees and included 179 with suspected COVID-19, but who had mild COVID and who had not been admitted to hospital with COVID, and 122 without suspected COVID-19. Persistent fatigue was present in 35% (49/142) of the suspected COVID-19 group and 13% (14/109) of the non-COVID-19 group (odds ratio 3.65; 95% confidence interval = 1.82 to 7.32). Prognostic factors for persistent fatigue included low education level, absence of a partner, high neuroticism (using the Eysenck Personality Questionnaire Revised-Short Form), low resilience, high frequency of GP contact, medication use, and threatening experiences in the past. The latter three factors appeared to be prognostic factors for persistent fatigue specifically after COVID-19 infection.

**Conclusion:**

GP patients with COVID-19 (who were not admitted to hospital with COVID) have a fourfold higher chance of developing persistent fatigue than GP patients who had not had COVID-19. This risk is even higher in psychosocially vulnerable patients who had COVID-19.

## INTRODUCTION

During the first wave of the COVID-19 crisis, uncertainty regarding diagnosis, prognosis, testing, and treatment caused anxiety in the population. Although some patients with COVID-19 remained asymptomatic, others experienced symptoms such as coughing, fever, and myalgia; others had to be admitted to the hospital or even the intensive care unit.^1^^,^^2^ Although the majority of people with COVID-19 recovered completely, it was anticipated that some patients would have persistent symptoms such as fatigue, cough, and depression.^3^ This persistent presence of illness is called long COVID and it significantly impairs the quality of life of patients.^4^ The World Health Organization defines long COVID as a condition that *‘occurs in individuals with a history of probable or confirmed SARS-CoV-2 infection, usually 3 months from the onset of COVID-19 and with symptoms that last for at least 2 months and cannot be explained by an alternative diagnosis’*.^5^ Fatigue is the most common symptom associated with long COVID.^6^ Post-infection chronic fatigue and other persistent complaints are also observed in other infectious diseases, such as Lyme disease and Q fever.^7^^–^9

The anticipated scenario of long-lasting symptoms after COVID-19 has proven true and a few factors, such as female sex and older age, seem to be associated with persistent symptoms.^10^^–^15 However, other sociodemographic factors, such as socioeconomic and marital status, have not been studied. Some lifestyle factors, such as high body mass index (BMI) and smoking, were found to be associated with persistent symptoms after COVID-19 in a few studies, whereas others did not find this association.^13^^–^16 There is a lack of information on the associations between other lifestyle factors and long COVID. For multiple chronic and psychiatric comorbidities, inconsistent results have been reported.^11^^,^^16^^,^^17^ For other post-infection complaints, associations with psychosocial factors have been noted.^18^ This suggests that psychosocially vulnerable patients might have a higher risk of developing persistent symptoms after COVID-19; however, this has not yet been investigated.

Most studies investigating COVID-19 have focused on the acute phase, but interest in long COVID has also increased. However, often only patients admitted to hospital are included in studies of long COVID, whereas the majority of people with COVID-19 present with mild symptoms and are managed in primary care.^2^ Because of the focus on patients with severe infections and inconsistencies in previous research, little is known about persistent symptoms and their prognostic factors in patients with mild COVID-19 attending primary care. Therefore, the aim of this study was to determine the prevalence of persistent fatigue after COVID-19 in patients with mild infection, compared with age- and sex-matched patients who consulted their GP for non-COVID-19-related symptoms during the same period and to identify the prognostic factors for persistent fatigue.

**Table:** How this fits in

Little is known about persistent fatigue and its prognostic factors in patients with mild COVID-19 who are attending primary care. Insight into persistent fatigue after COVID-19 infection and its prognostic factors can make GPs aware of the increased risk of persistent fatigue in specific patients and allow them to adapt treatment plans accordingly. This study found that patients suspected of having COVID-19 had an almost fourfold higher risk of persistent fatigue than patients without COVID-19. Low educational level, absence of a partner, high neuroticism, low resilience, high frequency of GP contact, medication use, and threatening experiences in the past were important prognostic factors for persistent fatigue. This suggests that psychosocially vulnerable patients are at a higher risk of developing persistent fatigue after COVID-19 infection.

## METHOD

### Study population

A prospective cohort study was performed and is reported according to STROBE guidelines.^19^ Patients were recruited from four Dutch GP practices in the Nijmegen region (east of the Netherlands). These practices are members of the family medicine network (FaMe-Net), which is a network of GPs with years of experience in registration and coding according to the International Classification of Primary Care (ICPC).^20^ Patients from these practices understand that their electronic health records (EHRs) may be used for scientific research.

At the very beginning of the pandemic, when there was no test capacity, FaMe-Net used a specific code (ICPC R83) for patients with a suspicious clinical picture of COVID-19.^21^ All patients with the ICPC code R83 between 1 March and 31 May 2020 were invited to participate. For every patient with suspected COVID-19, a matched non-COVID-19 patient was invited to participate. The participants were informed that the aim of this study was to investigate the long-term health status of people who had experienced COVID-19 compared with people who had not. They were matched based on sex, age (plus or minus 3 years), and GP practice. The patients had to be at least 18 years of age. The matched non-COVID-19 patients visited the GP during the same period for any complaint, except respiratory complaints or fever. The participants provided informed consent and were aware of the subject of the study.

### Data collection

The patients received online questionnaires approximately 3, 6, and 15 months after contacting their GP. Using online questionnaires, information on the outcomes and prognostic factors were obtained. In addition, patients were asked if they had become infected with COVID-19 during the study period. Participants in both the suspected COVID-19 group and the non-COVID-19 group who tested positive for COVID-19 during the 15-month follow-up continued to be included in the main analysis. Additional patient information regarding potential prognostic factors was collected from patients’ EHRs.

### Measures

The primary outcome in this study was persistent fatigue, measured using the Checklist of Individual Strength 8R (CIS8R). This standardised and validated questionnaire has been used for healthy individuals as well as those with respiratory diseases.^22^ The CIS8R has good internal consistency (Cronbach’s alpha of 0.92 in patients with rheumatoid arthritis) and good reproducibility (intraclass correlation coefficient 0.81).^23^ The severity of fatigue was measured with eight questions about the intensity of fatigue in the past 2 weeks on a seven-point Likert scale. The sum score ranges from 8 to 56, which is categorised as ‘no fatigue’ (CIS8R sum score <27) or ‘fatigue’ (CIS8R sum score ≥27).^22^^,^^24^

Based on fatigue scores at 3, 6, and 15 months, the primary outcomes were defined as follows:

- ‘no persistent fatigue’: ‘no fatigue’ scored on at least one of the follow-ups; and
- ‘persistent fatigue’: ‘fatigue’ scored on all three follow-ups or at two follow-ups with missing data from the other occasion.

Patients who reported ‘fatigue’ at one follow-up but did not participate on both other occasions, or those who did not participate in any of the surveys, were coded as ‘missing’.

Based on what was known about other post-infectious diseases, information was also collected on sociodemographic, lifestyle, and vulnerability factors. Sociodemographic factors included sex, age, education level, and marital status. Lifestyle factors included BMI, smoking, and alcohol use. Vulnerability factors included ‘neuroticism’, life events, resilience, perceived personalised GP care, comorbidities, medication use, and frequency of contact with a GP.^25^

Neuroticism was measured using the Eysenck Personality Questionnaire Revised-Short Form (EPQR-S),^26^ life events using the Brugha questionnaire,^27^ resilience using the Sense of Coherence-13 (SOC-13) questionnaire,^28^ and perceived personalised care provided by their GP using the Person Centered Primary Care Measure (PCPCM).^29^ Higher scores indicated higher levels of neuroticism, more life events, stronger resilience, and higher perceived personalised care from their GP.

Data on smoking, alcohol use, comorbidities, medication use, and frequency of contact with a GP were collected from the EHR. For contact frequency, the number of contacts with a GP in the year before inclusion in the study was used. This variable was made dichotomous, using a cut-off of ≥13 contacts, as this was the 80th percentile. For the number of medications, all active medications were summed. These included all the Anatomical Therapeutical Classification codes that were used during the inclusion period (from 1 March 2020 to 31 May 2020). Medication use was also transformed into a dichotomous variable, using a cut-off of ≥5 medications, which is often used to define polypharmacy. For comorbidities, all patients’ chronic comorbidities were summed (Supplementary Box S1).

### Analysis

The data were cleaned and analysed using RStudio.^30^ Using a multilevel generalised mixed-effects logistic regression (glmer) analysis, the odds ratios (OR) and corresponding 95% confidence intervals (CIs) for the association between the groups (suspected COVID-19 or non-COVID-19) and persistent fatigue were calculated. Levels were added for GP practice and matched patien31t-pairs to correct for clustering and matching. A sensitivity analysis was performed, excluding patients in the non-COVID-19 group who tested positive during the study. Thereafter, univariate multilevel analysis was performed in the suspected COVID-19 group only to determine prognostic factors for persistent fatigue. Individuals with missing data were excluded analysis by analysis. Spearman’s rho was used to check the correlation between the statistically significant prognostic factors.

Multivariable multilevel analysis (forward conditional) was performed, including significant prognostic factors from the univariate analyses, after checking for multicollinearity. Finally, the analysis was performed in the whole study population with interaction terms between the groups and prognostic factors to determine whether the associations between the prognostic factors and persistent fatigue differed between the groups. In the case of significant interaction effects, stratum-specific ORs were calculated and presented. For the multilevel models a *P*-value of 0.05 was used as a cut-off. For the interaction terms a less strict *P*-value of 0.10 was used.^31^

## RESULTS

### Study population

**Figure 1.**
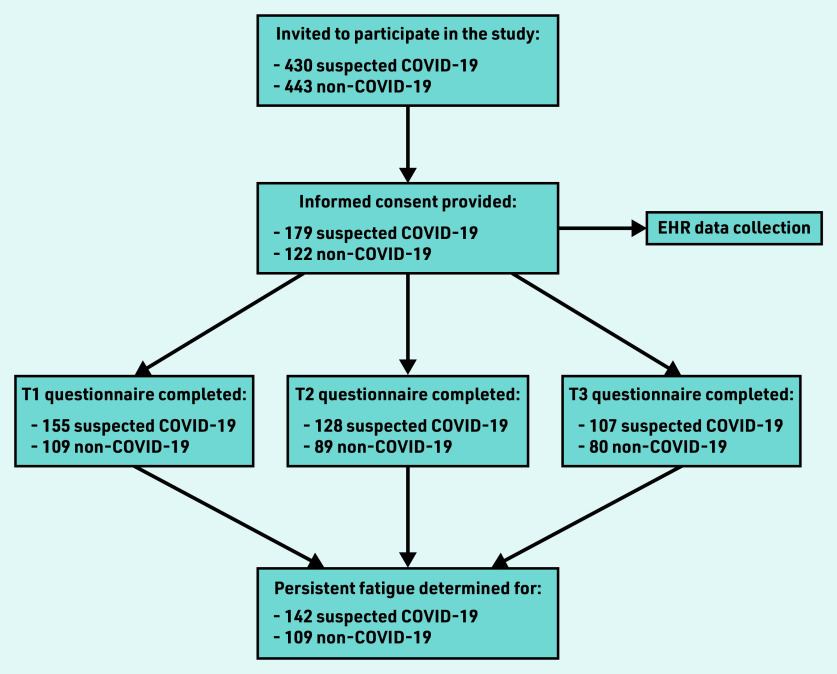
*Flowchart of participants’ inclusion and follow-up. EHR = electronic health record. T1 = time one (3 months). T2 = time two (6 months). T3 = time three (15 months).*

A total of 430 patients with suspected COVID-19 and 443 patients without suspected COVID-19 were invited to participate. Of these, 301 patients participated: 179 (42% of 430) in the suspected COVID-19 group and 122 (28% of 443) in the non-COVID-19 group. In the suspected COVID-19 group, 28 patients had confirmed COVID-19 from a positive polymerase chain reaction test, whereas the rest were not tested. In the non-COVID-19 group, nine patients tested positive for COVID-19 during the study. The response rates were 88% (*n* = 264), 72% (*n* = 217), and 62% (*n* = 187) for the 3-, 6-, and 15-month follow-up periods, respectively (Figure 1).

All baseline characteristics were comparable between the suspected COVID-19 and non-COVID-19 groups (*P*>0.05) (Table 1).

**Table 1.** Baseline characteristics in the suspected COVID-19 group and non-COVID-19 group (*n* = 301)

**Characteristics**	**Suspected COVID-19 group (*n* = 179)**	**Non-COVID-19 group (*n* = 122)**	***P*-value**
**Sex, *n* (%)**			0.296
Males	62 (35)	50 (41)	
Females	117 (65)	72 (59)	

**Age, years, mean (SD)**	47.3 (12.9)	48.6 (13.2)	0.388

**BMI,^a^ *n* (%)**			0.564
Normal weight	82 (55)	62 (59)	
Overweight/obese	66 (45)	43 (41)	

**Education,^a^ *n* (%)**			
High	95 (62)	68 (63)	0.730
Moderate	42 (27)	26 (24)	
Low	16 (11)	14 (13)	

**Marital status,^a^ *n* (%)**			0.546
Partner currently	120 (78)	88 (81)	
No partner currently	33 (22)	20 (19)	

**Smoking,^a^, *n* (%)**			0.928
No or quit before 2015	111 (85)	79 (85)	
Yes or quit since 2015	19 (15)	14 (15)	

**Alcohol use,^a^ *n* (%)**			0.069
No (or quit before 2015)	24 (18)	9 (10)	
Yes (or quit since 2015)	106 (82)	84 (90)	

**Number of chronic comorbidities,*n* (%)**			0.060
None	28 (16)	12 (10)	
One	29 (16)	32 (26)	
>1	122 (68)	78 (64)	

**Frequency of GP contact, *n* (%)**			0.723
<13	141 (79)	94 (77)	
≥13	38 (21)	28 (23)	

**Number of medications, *n* (%)**			0.340
<5	137 (77)	99 (81)	
≥5	42 (23)	23 (19)	

**Neuroticism,^a^ mean (SD)**	3.6 (3.5)	3.3 (3.4)	0.434

**Life events (last 12 months),^a^ mean (SD)**	1.1 (1.4)	0.8 (1.0)	0.074

**Life events (earlier),^a^ mean (SD)**	3.7 (2.5)	3.5 (2.5)	0.662

**Resilience,^a^ mean (SD)**	69.5 (13.6)	70.8 (12.9)	0.441

**Perceived personalised care from GP,^a^ mean (SD)**	3.1 (0.6)	3.2 (0.5)	0.151

a *For the suspected COVID-19 and non-COVID-19 groups, there were missing data for BMI (*n *= 31 and 17), education (*n *= 26 and 14), marital status (*n *= 26 and 14), smoking (*n *= 49 and 29), alcohol use (*n *= 49 and 29), neuroticism (*n *= 35 and 22), life events (*n *= 38 and 25), resilience (*n *= 36 and 22), and for perceived personalised care from GP (*n *= 40 and 26). BMI = body mass index. SD = standard deviation.*

#### Non-participants

Males comprised 36% and 35% of the suspected COVID-19 and non-COVID-19 non-participants, respectively. The mean age of the non-participants in both the suspected COVID-19 and non-COVID-19 groups was 42 years (SD 14).

### Fatigue at 3, 6, and 15 months

**Figure 2.**
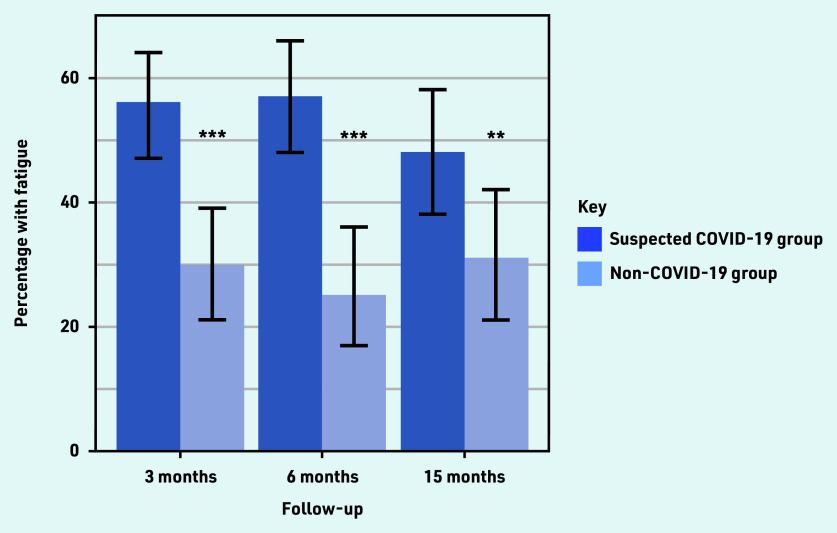
*Percentage of patients classified as fatigued at 3, 6, and 15 months in the suspected COVID-19 and non-COVID-19 groups. Data on fatigue were not available for all patients on all occasions. In the suspected COVID-19 group the number with missing data at 3, 6, and 15 months were 32, 54, and 73, respectively. In the non-COVID-19 group the number with missing data at 3, 6, and 15 months were 17, 35, and 44, respectively.* *P *<0.05.* **P *<0.01.* ***P *<0.001.*

A total of 56%, 57%, and 48% of the suspected COVID-19 group were classified as fatigued, compared with 30%, 25%, and 31% in the non-COVID-19 group at 3, 6, and 15 months, respectively (Figure 2). Differences between the groups were statistically significant at all three time points.

### Persistent fatigue

Among the suspected COVID-19 group, 35% were defined as having persistent fatigue at the end of follow-up, whereas this was only 13% in the non-COVID-19 group. People suspected of having COVID-19 had 3.65 times higher odds (95% CI = 1.82 to 7.32) of persistent fatigue than those in the non-COVID-19 group. The sensitivity analysis, which excluded non-COVID-19 patients who tested positive during follow-up, showed similar results (data not shown).

In the suspected COVID group, people with a lower level of education were more often persistently fatigued than those with a higher level of education (OR 3.13) (Table 2). Furthermore, those who did not have a partner showed a higher risk of persistent fatigue (OR 3.25).

**Table 2.** Prognostic factors for persistent fatigue in the suspected COVID-19 group only — results of univariate logistic multilevel models

**Characteristic**	**Persistent fatigue (*n* = 49)**	**No persistent fatigue (*n* = 93)**	**OR (95% CI)**
**Sex, *n* (%)**			
Males	14 (29)	42 (45)	Reference
Females	35 (71)	51 (55)	2.06 (0.98 to 4.32)

**Age, years, mean (SD)**	49.6 (12.8)	47.7 (13.0)	1.01 (0.98 to 1.04)

**BMI, *n* (%)**			
Normal weight	21 (44)	54 (61)	Reference
Overweight/obese	27 (56)	35 (39)	1.98 (0.98 to 4.04)

**Education,^a^ *n* (%)**			
High	25 (52)	61 (68)	Reference
Moderate	14 (29)	22 (24)	1.55 (0.68 to 3.50)
Low	9 (19))	7 (8)	3.13 (1.06 to 9.68)

**Marital status,^a^ *n* (%)**			
Partner currently	32 (67)	78 (87)	Reference
No partner currently	16 (33)	12 (13)	3.25 (1.38 to 7.64)

**Smoking,^a^, *n* (%)**			
No/quit before 2015	35 (90)	60 (88)	Reference
Yes/quit since 2015	4 (10)	8 (12)	0.86 (0.24 to 3.05)

**Alcohol use,^a^ *n* (%)**			
No (or quit before 2015)	11 (28)	10 (15)	Reference
Yes (or quit since 2015)	28 (72)	58 (85)	0.44 (0.17 to 1.16)

**Number of chronic comorbidities,*n* (%)**			
None	5 (10)	17 (18)	Reference
1	5 (10)	19 (20)	0.89 (0.22 to 3.63)
>1	39 (80)	57 (61)	2.33 (0.79 to 6.83)

**Frequency of GP contact, *n* (%)**			
<13	33 (67)	79 (85)	Reference
≥13	16 (33)	14 (15)	2.74 (1.20 to 6.24)

**Number of medications, *n* (%)**			
<5	30 (61)	79 (85)	Reference
≥5	19 (39)	14 (15)	3.57 (1.59 to 8.02)

**Neuroticism,^a^ mean (SD)**	5.2 (4.0)	2.7 (2.9)	1.24 (1.11 to 1.38)

**Life events (last 12 months),^a^ mean (SD)**	1.4 (1.6)	0.9 (1.2)	1.32 (1.01 to 1.73)

**Life events (earlier),^a^ mean (SD)**	4.1 (2.3)	3.7 (2.5)	1.08 (0.93 to 1.25)

**Resilience,^a^ mean (SD)**	63.6 (15.4)	72.9 (11.2)	0.95 (0.92 to 0.98)

**Perceived personalised care of the GP,^a^ mean (SD)**	3.1 (0.7)	3.1 (0.5)	0.81 (0.43 to 1.54)

a *There were missing data for BMI (*n *= 1 and 4), education (*n *= 1 and 3), marital status (*n*= 1 and 3), smoking (*n *= 10 and 25), alcohol use (*n *= 10 and 25), neuroticism (*n *= 2 and 5), life events (*n *= 3 and 7), resilience (*n*= 2 and 6), and perceived personalised care of GP (*n *= 4 and 8). BMI = body mass index. CI = confidence interval. OR = odds ratio. SD = standard deviation.*

In addition, a high frequency of contact with a GP in the year before COVID infection (OR 2.74) and polypharmacy at time of inclusion (OR 3.57) were statistically significant prognostic factors. The same was observed for neuroticism (OR 1.24); people with a higher neuroticism score had greater odds of persistent fatigue after suspected COVID-19. Other prognostic factors significantly associated with persistent fatigue were the number of life events in the previous year (OR 1.32) and low resilience (OR 0.95). Females seemed to have higher odds of persistent fatigue than males (OR 2.06), as did overweight/obese patients (OR 1.98), but these associations were borderline not-significant. Smoking, alcohol use, comorbidities, earlier life events, and perceived personalised GP care did not seem to be associated with persistent fatigue after COVID-19.

In a multivariable analysis, the effects of neuroticism (OR 1.22, 95% CI = 1.09 to 1.36) and number of medications (OR 3.22, 95% CI = 1.30 to 7.96) remained significant, indicating that these were independently related to persistent fatigue. The model explained 22.4% of the variance in persistent fatigue (Nagelkerke R-squared = 0.224). Exploring the correlations between the significant prognostic factors revealed that neuroticism and resilience were strongly correlated (Spearman’s rho = −0.67, *P*<0.001), whereas frequency of contact with a GP and medication use showed a moderate correlation (Spearman’s rho = 0.42, *P*<0.001). Therefore, the multivariate model did not include resilience and frequency of GP contact. In addition, life events, education, and marital status did not add significantly to the multivariable model.

The statistical significance of the interaction term between groups (suspected COVID-19 or non-COVID-19) and potential prognostic factors was assessed. Table 3 shows the prognostic factors for both the suspected COVID-19 and non-COVID-19 groups, for which this interaction term was statistically significant. Age was found to be linearly associated with persistent fatigue, with the percentage decreasing with increasing age category. Higher age was found to be a protective factor for persistent fatigue in the non-COVID-19 group (OR 0.94). However, there was no association between age and persistent fatigue in the suspected COVID-19 group (OR 1.01). In contrast, a higher frequency of contact with a GP in the year before COVID-19 infection (OR 2.74), higher number of medications (OR 3.57), and more life events (OR 1.32) were specific prognostic factors for persistent fatigue in the suspected COVID-19 group but not in the non-COVID-19 group (Table 3).

**Table 3.** The results of the logistic multilevel models of the prognostic factors for persistent fatigue in the suspected COVID-19 and non-COVID-19 groups for which the interaction term was significant

**Factor**	**Suspected COVID-19 group, OR (95% CI) (*n* = 142)**	**Non-COVID-19 group, OR (95% CI) (*n* = 109)**	***P*-value for interaction term with group^a^**
**Age**	1.01 (0.98 to 1.04)	0.94 (0.90 to 0.99)	0.010

**Frequency of GP contact**			
< 13	Reference	Reference	—
≥13	2.74 (1.20 to 6.24)	0.49 (0.10 to 2.36)	0.052

**Number of medications**			
<5	Reference	Reference	—
≥5	3.57 (1.59 to 8.02)	0.67 (0.14 to 3.23)	0.056

**Life events (last 12 months)^b^**	1.32 (1.01 to 1.73)	0.49 (0.20 to 1.18)	0.040

a P*-value <0.10 was considered a significant interaction.*

b *Data on life events were missing in 38 and 25 in the suspected COVID-19 group and non-COVID-19 group, respectively. CI = confidence interval. OR = odds ratio.*

## DISCUSSION

### Summary

This prospective cohort study investigated the prevalence of and prognostic factors for persistent fatigue after the first wave of COVID-19. Patients suspected of having COVID-19 appeared to have an almost fourfold higher risk of persistent fatigue than those without COVID-19. This study found that, of the patients who contacted their GP during the first wave of the pandemic, an increased risk of persistent fatigue was seen in those who:

- had a lower educational level;
- had no partner;
- had more frequent contact with a GP in the year before COVID-19 infection;
- used ≥5 medications;
- had higher scores on neuroticism;
- had lower scores on resilience; and
- had more life events in the past 12 months.

The effects of neuroticism and number of medications were independently related in a multivariable analysis; these factors explained 22% of the variance in persistent fatigue. After COVID-19 infection, the frequency of contact with a GP in the year before COVID-19 infection, medication use, and life events in the past 12 months were specific prognostic factors for the occurrence of persistent fatigue. These results seem to indicate that psychosocially vulnerable patients are more likely to report persistent fatigue after COVID-19 infection.

### Strengths and limitations

An important strength of this study was the availability of a matched control group. First, this enhances comparability and reduces bias because of confounding factors. Second, the prevalence of persistent fatigue in the control group (13%) refined the interpretation of the prevalence in the suspected COVID-19 group (35%). In addition, the inclusion of this control group made it possible to discriminate between prognostic factors for persistent fatigue in general and prognostic factors specifically in the suspected COVID-19 group. Another strength is that participants were selected from multiple practices, where GPs had extensive experience in coding multiple aspects of every contact with each patient. This enabled the integration of reliable information from the EHR. Another strength is the prospective measurement of fatigue at three time points, which provides more reliable data than retrospective measurements. In addition, the classification of persistent fatigue is rather strict. When someone was classified as not fatigued at only one time of measurement, the person was classified as having no persistent fatigue. Another strength of this study is the long-term follow-up of 15 months.

The most important limitation was that the diagnoses of COVID-19 were made by the GP, since tests were not accessible to the general public in the Netherlands at the beginning of the pandemic. Therefore, it is uncertain whether these patients actually had COVID-19. However, because of lockdown measures there were scarcely any other infections at that time,^21^ making it very likely that these patients had COVID-19. Moreover, because of this method of diagnosis it was possible to start this study during the first wave and thus record long-term outcomes relatively soon. Another limitation was the possibility that patients in the non-COVID-19 group had COVID-19 either before or after study entry. However, to check for this, the patients were asked if they had COVID-19, and the sensitivity analysis, excluding these people, revealed that this did not substantially change the results.

Another limitation was the lack of baseline measures. It is known that patients typically have more symptoms than what is recorded in EHRs, and this is certainly true in the case of fatigue.^32^ Therefore, it is possible that there were already differences in the outcomes before this study. Care should be taken before concluding that the prevalence of persistent fatigue in the suspected COVID-19 group can be attributed completely to COVID-19 infection. The small group of patients with suspected COVID-19 can also be considered a limitation.

Finally, the participants in this study were told that the aim of the study was to investigate the long-term health status of people who had experienced COVID-19 compared with people who had not. Therefore, they were aware that this study was about COVID-19, making it more likely that they are attentive to COVID-19 and related complaints.

### Comparison with existing literature

According to the available literature, female patients seem to have a higher risk of developing persistent symptoms after COVID-19.^12^^–^15^,^^33^ In the current study, female sex was not significantly associated with persistent fatigue after COVID-19 (OR 2.06, 95% CI = 0.98 to 4.32). A similar result was observed for obesity. According to previous literature, obesity is associated with more serious disease and persistent symptoms after COVID-19;^13^^–^15 however, the current study found no significant association between high BMI and persistent fatigue (OR 1.98, 95% CI = 0.98 to 4.04). It is possible that the association between obesity and persistent fatigue after a severe COVID-19 infection is different compared with that after milder infections that do not lead to hospital admission.

Some studies have found that chronic comorbidities are risk factors for persistent symptoms after COVID-19.^11^ In the current study, this association was not observed. However, a statistically significant association was observed between the number of medications used and persistent fatigue. As it is likely that medication use and comorbidities correlate with each other, this might be an indication that comorbidities play a role. In addition to the number of medications taken, the current study identified several other prognostic factors. Neuroticism, life events, marital status, resilience, education level, and prior frequency of contact with a GP were significantly associated with persistent fatigue after COVID-19. The effects partly overlap, and the effects of neuroticism and the number of medications were found to be independently related to persistent fatigue. These prognostic factors have not been described in previous studies of long COVID. However, some studies suggest that psychosocial vulnerability and low income are associated with symptoms of long COVID, which is confirmed by the results of the current study.^34^^,^^35^

### Implications for research and practice

Further research is needed to determine how symptoms of long COVID develop. As it is a new disease it is unpredictable whether the symptoms will eventually reduce. Further studies are needed to confirm the prognostic factors identified in this study.

The results of this study show that psychosocially vulnerable people have a higher risk of persistent fatigue after COVID-19 infection. Therefore, psychosocial factors should be considered when studying diseases and their risk factors. With regard to long COVID, the results of this study can be used in clinical practice. When a patient has COVID-19 infection and meets some of the prognostic psychosocially vulnerable criteria, the GP should be aware of the increased risk of persistent fatigue and adapt the treatment plan to prevent its development.

In conclusion, the risk of persistent fatigue is almost four times higher in patients with suspected COVID-19 than in those with no history of COVID-19. Furthermore, the risk of occurrence of persistent fatigue is heightened by several psychosocial vulnerability factors.

## Data

Data will be made publicly available in the Radboud data repository once all follow-ups are completed.
